# Highly selective and sensitive probes for the detection of Cr(vi) in aqueous solutions using diglycolic acid-functionalized Au nanoparticles†

**DOI:** 10.1039/c9ra00010k

**Published:** 2019-04-08

**Authors:** Yang Zhang, Ruixi Bai, Zhigang Zhao, Qiuxia Liao, Peng Chen, Wanghuan Guo, Chunqing Cai, Fan Yang

**Affiliations:** CAS Key Laboratory of Design and Assembly of Functional Nanostructures, Fujian Provincial Key Laboratory of Nanomaterials, Fujian Institute of Research on the Structure of Matter, Chinese Academy of Sciences Xiamen 361021 China yzhang@fjirsm.ac.cn fanyang2013@fjirsm.ac.cn; Xiamen Institute of Rare Earth Materials, Haixi Institute, Chinese Academy of Sciences Xiamen 361021 China

## Abstract

In this study, a variety of diglycolic acid-functionalized gold nanoparticle (Au NP) probes are reported, which are highly sensitive for the detection of chromium ions, Cr(vi) ions, at low concentrations in aqueous solutions based on the application of surface plasmon resonance (SPR) theory. Due to its outstanding affinity for Cr(vi) ions, the capped diglycolic acid would induce the aggregation of the NP probes upon encountering them; this was evidenced by the obvious red-shifting of the SPR peak and the enlarged size of the NPs. For the same reason, the selectivity of the probe for Cr(vi) against other heavy metal ions was found to be remarkable. Under optimized conditions, the probe showed the limit of detection (LOD) of 0.32 ppb for Cr(vi) and a linear detection scale ranging from 0.32 ppb to 0.1 ppm. To the best of our knowledge, this is probably the lowest LOD reported for Cr(vi) detection among those of the methods based on SPR.

## Abstract 2

In this study, we proposed a diglycolic acid-functionalized gold nanoparticle (Au NP) probe, which are highly sensitive for the detection of chromium ions based on the application of surface plasmon resonance (SPR) theory.

## Graphical abstract

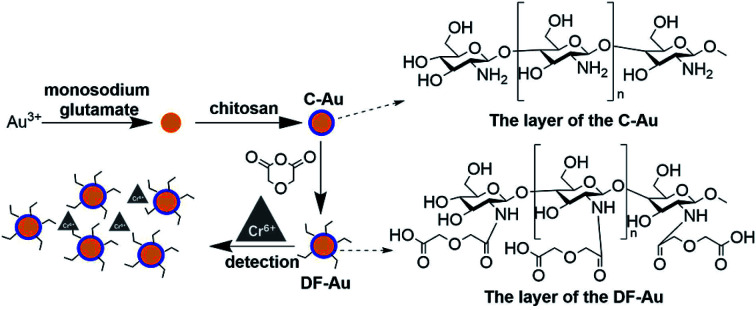

## 1. Introduction

Due to its large-scale applications in metallurgy, chemical engineering, steel making and many other areas, chromium is extensively discharged from the industries; this poses a severe threat to the environment and human health.^1–3^ Compared with trivalent chromium, which is an essential nutrient for humans, hexavalent chromium is a lethal toxicant to human beings.^4–6^ According to the rules of the United States Environmental Agency, the upper limit of Cr(vi) in drinking water is 0.05 ppm. As reported, the overdose of Cr(vi) can cause fatal diseases ranging from ulceration to cancer.^7–9^ Therefore, the detection of Cr(vi) at trace amounts in water is extremely necessary and has drawn worldwide concern for a long time.^10–13^

Due to the detrimental effects of chromium on the environment and bio-health, diverse analytical techniques, such as atomic adsorption spectrometry, chromatography, inductively coupled plasma-mass spectrometry (ICP-MS), and spectrophotometry, have been proposed for the detection of Cr(vi) in aqueous solutions. However, these strategies have numerous intrinsic drawbacks such as the requirement of expensive instruments, complex pretreatment, and sophisticated operation. To circumvent these problems, some convenient and efficient detection strategies have been established such as fluorescence,^14–18^ electrochemical,^19–21^ and surface plasmon resonance (SPR) methods.^22,23^

Among these solutions, the sensors based on the SPR technique have received significant attention due to their characteristic superiorities such as convenience, high sensitivity, rapidity, and bio-friendliness.^21,24–27^ They are triggered by a physical contact or a chemical reaction of the analyte with the surface of the sensors, usually in the forms of nanoparticles or thin films; this causes shifts of the plasmon resonance peaks. This has been regarded as an ideal approach for the detection of pesticides, toxic gases, drug molecules, and hazardous heavy metals.^28–32^ The common plasmon-generating substrates in this method are the nanostructures of gold, silver, and platinum.^33–35^ With moderate activity and cost as well as adjustable morphologies and properties, gold nanoparticles (Au NPs) are the most convenient and commonly used substrates in the SPR methods.^29,36,37^

As the naked Au NPs or those encircled by only surfactants show limited sensitivity, functionalization is often implemented to enhance their sensitivity.^38–40^ To date, some effective functionalization routes, such as chemical grafting or coating, have been implied to endow additional superiorities, such as better selectivity or higher sensitivity, to the probes.^41–45^ Nowadays, a modifying agent having significantly selective binding tendency towards the analyte than the other ions is required.

In this study, a sensitive SPR detection system for Cr(vi) based on diglycolic acid-functionalized Au NPs was established. For a long time, diglycolic acid and the derived structures have drawn the attention of researchers due to their outstanding selectively coordinative properties for some rare earth elements and heavy metals that are probably generated due to particular steric hindrance. The as-established probing system shows the remarkable LOD of 3.2 ppb for Cr(vi), which, to the best of our knowledge, is probably the lowest value among those of the SPR sensors. There is a linear relationship between the decrement of the intensity of the SPR peak and the concentration of Cr(vi) in the range from 3.2 ppb to 0.1 ppm; this makes the application of the proposed system possible for the quantitative measurement of Cr(vi) in water. Moreover, the selectivity of this system towards other ions, including Cr(iii), is relatively high; based on these findings, the concentration of Cr(vi) in some practical samples has also been tested in this study.

## 2. Experimental

### 2.1 Chemicals

Chloroauric acid, monosodium glutamate, chitosan, diethylene glycol, sodium hydroxide, and hydrochloric acid were purchased from Aladdin Bio-chemical Reagent Co., Ltd (Shanghai, China). All the reagents were of analytical degree and used directly without further purification. Ultrapure water was applied throughout the entire experiment.

### 2.2 Instruments and measurements

TEM images were obtained by a scanning electron microanalyzer (Hitachi SU1510). FTIR spectroscopy was conducted using the Nicolet iS 50 Fourier transform infrared spectrometer. The UV-vis spectra were obtained using the Agilent Cary 5000 spectrophotometer. The DLS and *ζ*-potential data were acquired using the Brookhaven Omni size/potentiometric analyzer. The referenced detection of Cr(vi) in real water samples was carried out using the Agilent 8800 inductively coupled plasma tandem mass spectrometer (ICP-MS/MS).

### 2.3 Preparation of the chitosan-capped unfunctionalized Au NPs (C-Au)

The synthetic protocol was based on a reference method.^46^ Typically, 100 ml of 0.1 mM HAuCl_4_ was heated to boiling. On boiling, 12.68 mg of monosodium glutamate was added to it under stirring. Stirring was continued for about 10 minutes until the solution turned deep red. Then, the solution was quickly quenched to room temperature. While quenching, 10 ml 30% chitosan solution in 1% HCl was added to the system.

### 2.4 Preparation of the diglycolic acid-functionalized Au NPs (DF-Au)

As illustrated in Scheme S1,† the diglycolic acid groups were modified on the surfaces of C-Au through an anhydride ring-opening process as follows. At first, the abovementioned aqueous solution was freeze-dried for 24 h at −40 °C to obtain the C-Au particles. After this, the C-Au particles and some amount of diglycolic anhydride (see Section 3.2) were dispersed in 100 ml DMF. Then, the mixture was heated for 48 h at 40 °C. After being washed several times with ultrapure water, the product was re-freeze dried for 24 h at −40 °C. Then, DF-Au was re-dispersed in 100 ml water.

## 3. Results and discussions

### 3.1 A comparison with C-Au

To clarify the mechanism of the sensitivity and selectivity of the as-prepared sensor, a comparison was made between our products and those capped with only chitosan (C-Au). According to an article reported by Sugunan *et. al.*, the purpose of the surface modification of Au NPs by pure chitosan is to distinguish the intensity of the UV-vis absorption peaks corresponding to the different concentrations of analytes.^46^ As reported by the authors, the capped chitosan could play the role of a coordinative shell sensitive to the concentration of analytes. However, pure chitosan, which is not a selective chelating agent, cannot endow selectivity to the probe. In this regard, to selectively detect Cr(vi) among many heavy metals, chitosan was initially functionalized by the DGA groups, which showed outstanding selectivity towards some heavy metals according to our published studies.^47^ According to the results, DF-Au exhibited much higher selectivity for Cr(vi) ions than C-Au (see Fig. 2); this suggested that an enhanced selectivity was endowed by functionalization. Moreover, *via* the prior functionalization step, the sensitivity of the sensor for Cr(vi) has been significantly enhanced.

### 3.2 Influence of the proportion of the functionalized amino groups of the capped chitosan

**Fig. 1:**
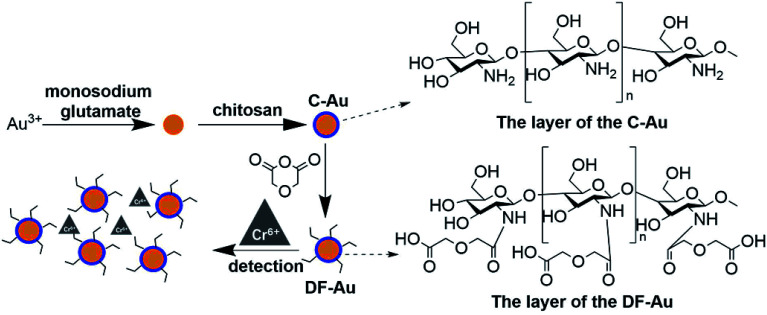
The schematic of the probe preparation and the sensing process.

Only a portion of the amino groups of chitosan was functionalized *via* the grafting of the DGA groups such that some primary amide sites remained and assisted the oxygen atoms to combinedly form a size- and charge-screen device. To comprehensively investigate the optimum proportion of the functionalized amines of chitosan, a batch of control experiments was conducted. *R*_d/c_ is defined as the molar ratio of diethylene glycol to chitosan added in the functionalization step. A series of *R*_d/c_ were used to examine the corresponding selectivity and sensitivity of the probe for Cr(vi). As illustrated in Fig. 3A, when the *R*_d/c_ was fixed at 0.6, both the selectivity and sensitivity were highest. This further proved that the primary amides played an essential role in assisting the oxygen atoms to form a highly selective and sensitive sensor for the detection of Cr(vi). Thus, all the following experiments were carried out with the *R*_d/c_ set at 0.6. To vividly illustrate the structures of the involved chemicals and the sensing mechanism, a schematic diagram is shown in Fig. 1.

### 3.3 Influence of the pH values

As revealed in some reported studies, the selective and coordinative ability of the DGA group is sensitive to the pH values. Therefore, different pH values (from 0.5 to 4.0) were used to find out the optimized pH condition for the following experiments using sodium hydroxide and hydrochloric acid as the pH-adjusting agents. It was found that the sensitivity of the Cr(vi) detection increased with the increasing pH values (see Fig. 3B); this was consistent with our earlier studies.^48,49^ However, as the heavy metal ions usually exist in acidic solutions and the pH value of 4.0 can result in the precipitation of some heavy metal ions and their interference with the performance of the sensors, the pH value has been fixed at 3.5 for the subsequent experiments.

**Fig. 2:**
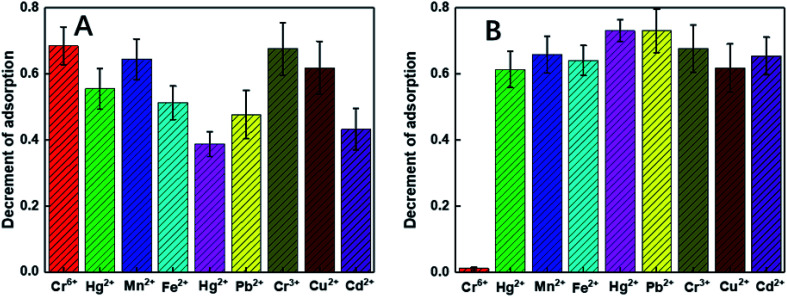
Selectivity controlled trials for (A) DF-Au and (B) C-Au towards different interferences (pH 3.5). Cr^6+^ is at the concentration of 0.1 ppm, others are at the concentration of 10 ppm. The vertical ordinate shows the decrement of the extinction peak at 517 nm.

**Fig. 3:**
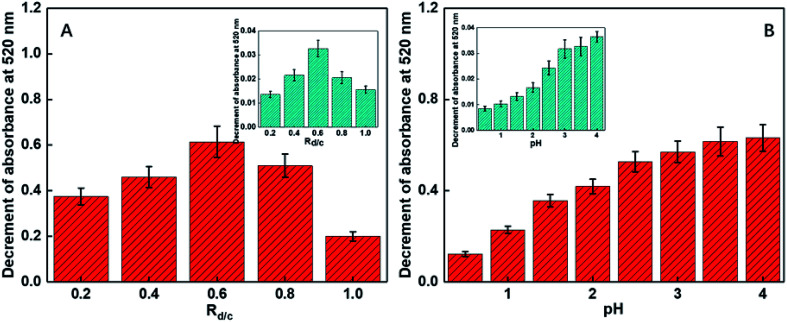
The schematic of the selectivity and sensitivity for Cr^6+^*versus* different values of (A) *R*_d/c_ and (B) pH. The concentration of Cr^6+^ was 0.1 ppm for the red columns, and 0.5 ppb for the insets. (The pH was set at 3.5 for (A), and the *R*_d/c_ was set at 0.6 for (B).)

### 3.4 Red shifting of the SPR peaks caused by the aggregation of Au NPs

**Fig. 4:**
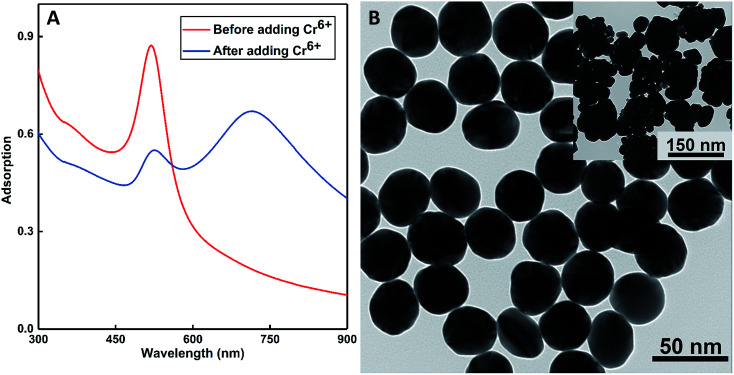
(A) The UV-vis extinctions of the as-prepared DF-Au sensors before and after the addition of 0.05 ppm Cr^6+^ and the color change from wine-red to purple (inset). (B) The TEM images of the as-prepared DF-Au sensors before and after the addition of 0.1 ppm Cr^6+^ (inset).

The as-prepared colloids displayed a wine-red color with the extinction peak at 520 nm in the UV-vis spectra (see the inset of Fig. 4A). After the addition of some amount of Cr(vi) (at the concentration of 0.1 ppm) to the solution, the color turned to purple at once; this was probably the indication of the aggregation of probes. This conjecture was confirmed by the red-shifting of the SPR peaks to 715 nm in Fig. 4A. More distinct results could be obtained from the TEM images shown in Fig. 4B: the as-prepared DF-Au displayed a uniform diameter of about 30 nm, whereas obvious aggregation could be observed after the addition of Cr(vi) ions, and the diameters increased to 130 nm (see the insets of Fig. 4B). Further characterizations of DLS and *ζ*-potential also certified the aggregation: the average diameter obtained from the DLS results was 28.22 ± 1.02 nm for the fresh probes and 140 ± 10.30 nm for the aggregated probes. In addition, the very small variance of these results illustrated that the sensors had a uniform size; this agreed well with the microscopy images. Furthermore, the freshly prepared DF-Au solution had a *ζ*-potential of −22.18 ± 2.84 mV, whereas the aggregated probes had a *ζ*-potential of −7.50 ± 0.87 mV, indicating that some degrees of sedimentation occurred with the introduction of Cr(vi).

### 3.5 Sensitive and selective detection of Cr(vi)

**Fig. 5:**
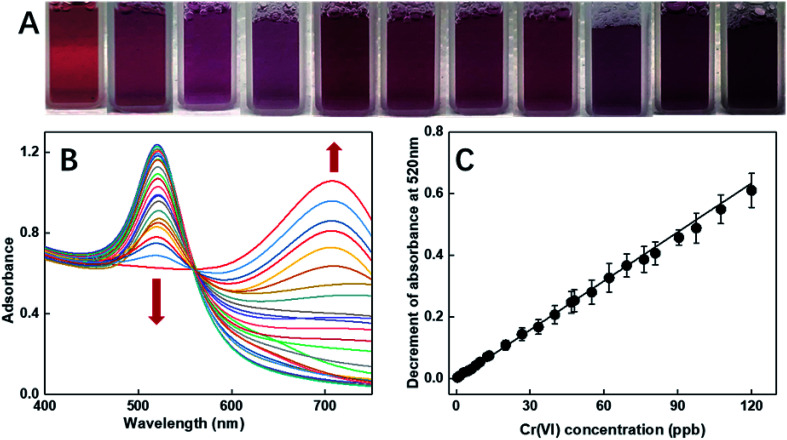
The (A) macroscopic colorimetric gradation and the (B) UV-vis extinctions of the DF-Au with different concentration of Cr(vi). The concentration of Cr(vi) increased from 0.32 ppb to 0.1 ppm from left to right in (A) and with the arrow direction in (B). The decrement of absorbance at 520 nm in the UV-vis extinctions *versus* the concentration of Cr(vi) (from 0.32 ppb to 0.1 ppm) is plotted in (C).

As illustrated in Fig. 2A, the selectivity of our probe for Cr(vi) among all the tested heavy metals ions (including Zn(ii), Hg(ii), Pb(ii), Cu(ii), Cd(ii), Fe(ii), Mn(ii), and Cr(iii)) was conspicuous even with the condition that the concentrations of the other ions were 100 times that of Cr(vi). To comprehensively explore the sensitivity of the probe, it was contacted with a series of Cr(vi) solutions with controlled concentrations ranging from 0.32 ppb to 0.1 ppm. Upon direct observation with naked eyes, we could see the color gradation of the reacted colloids from wine red to purple with the increasing concentration of the analytes (see Fig. 5A). Moreover, from the UV-vis spectra shown in Fig. 5B, it was depicted that the intensities of the peaks at 520 nm reduced gradually with an increase in the concentrations of the samples. In fact, the relationship between the decrement of the peak values and the corresponding concentrations of the samples could be fitted to a line (illustrated in Fig. 5C) with the coefficient quite near to 1, indicating good accuracy of the fitting. As shown, the LOD of the as-prepared probe for Cr(vi) is 0.32 ppb, which, to the best of our knowledge, is the lowest detection limit of colorimetric sensors reported to date.

The selectivity of the probe for Cr(vi) is probably due to the steric hindrance effect of the functionalizing agents of DGA, in which the oxygen and the nitrogen atoms in the carbonyl groups, the ether groups, the carboxyl groups and the amide groups consist a chelate-like structure.^50,51^ This system acted as a size- and charge-screening device. As Cr(vi) possesses smallest diameter and highest charge, which are preferred by the probe, it becomes the most sensitive ion among all the tested ions. As the carboxyl and amide groups chelate Cr(vi), which acts like a cross-linker, the DF-Au NPs are linked together, causing an agglomeration phenomenon, which has been confirmed by the abovementioned results.

To comprehensively compare this strategy with other existing methods, a comparison in the form of a table has been established in Table 1. We can conclude that this adsorbent has the merits of celerity, high selectivity, visualization (as the color would change upon its encounter with Cr(vi)), and low LOD.

**Table 1:** The comparison of the nature of some commonly used methods for the remediation of Cr(vi)

Strategies	Characteristics	Ref.
Ion exchange	High capacity, fast kinetics; low selectivity	54 and 55
Membrane adsorption	Spatial and time efficiency; membrane fouling, complicated operation	56 and 57
Biodegradation	Cost-efficiency; careful operation and selection of the bio-organisms	58 and 59
Electrochemistry methods	High capacity, cost-efficiency; sacrifice of anodes	60 and 61
Precipitating reduction	Easy operation, cost-efficiency; sludge production, consumption of reductants	62 and 63

### 3.6 Application for the detection of Cr(vi) in real water samples

For evaluating the applied performance of the as-synthesized sensor in the quantitative detection of Cr(vi) in real environmental water samples, samples of salt-lake water (from Qinghai Lake, China), sea waters (from Amoy, China and Ishikarihama, Japan respectively), and metallurgical industry wastewater (from Amoy, China, after decontamination) were reacted with the sensor to detect the contents of Cr(vi) in them. To adjust the concentrations of these samples to the working scale of our synthesized probe (0.32 ppb to 0.1 ppm), all the samples were diluted 10 times before conducting the test. As can be seen from the results displayed in Table 2, the Cr(vi) concentrations in the abovementioned real samples are 0.17 ppm, 0.04 ppm, 0.02 ppm, and 0.53 ppm. Furthermore, to verify the correctness of the results, the concentrations were measured using the reference method-ICP-MS, and the concentrations were found to be 0.15 ppm, 0.05 ppm, and 0.03 ppm, and 0.49 ppm, which matched quite well with those obtained using our newly developed sensor. In addition, the results corroborated well with those reported in some previous studies on some natural water samples.^52,53^

**Table 2:** The results of the quantitative Cr(vi) detection in real water samples using the as-prepared GF-Au sensor and the reference ICP-MS strategy

Waters & methods	GF-Au (ppm)	ICP-MS (ppm)
Salt-lake water	0.17 ± 0.022	0.15 ± 0.022
Amoy sea water	0.04 ± 0.031	0.05 ± 0.047
Ishikarihama sea water	0.02 ± 0.019	0.03 ± 0.027
Industrial waste	0.53 ± 0.046	0.49 ± 0.052

## 4. Conclusion

Overall, a functionalized chitosan-modified Au NP colorimetric sensor was synthesized and applied in the detection of hexavalent chromium among many heavy metals, including trivalent chromium, based on the SPR phenomenon. The colorimetric change could be easily detected by naked eyes or a UV-vis spectrophotometer. Compared with the sensor capped by pure chitosan, the newly developed sensor exhibited much better selectivity and sensitivity for hexavalent chromium. It is worth emphasizing that the as-prepared sensor shows a detection limit of 0.32 ppb of Cr(vi), which is the lowest value reported to date for a sensor using the SPR phenomena mechanism. Moreover, the chitosan used herein was initially partly functionalized through the grafting of DGA groups; this resulted in high selectivity and sensibility. Hence, this assay provides an efficient strategy of the functionalization of capping agents to improve the applied performance of the as-synthesized sensors.

## Conflicts of interest

There are no conflicts to declare.

## Supplementary Material

RA-009-C9RA00010K-s001

## References

[cit1] Barrera-Diaz C. E., Lugo-Lugo V., Bilyeu B. (2012). A review of chemical, electrochemical and biological methods for aqueous Cr(VI) reduction. J. Hazard. Mater..

[cit2] Wang H., Yuan X. Z., Wu Y., Zeng G. M., Chen X. H., Leng L. J., Wu Z. B., Jiang L. B., Li H. (2015). Facile synthesis of amino-functionalized titanium metal-organic frameworks and their superior visible-light photocatalytic activity for Cr(VI) reduction. J. Hazard. Mater..

[cit3] Han J. C., Chen G. J., Qin L. P., Mu Y. (2017). Metal Respiratory Pathway-Independent Cr Isotope Fractionation during Cr(VI) Reduction by Shewanella oneidensis MR-1. Environ. Sci. Technol. Lett..

[cit4] Zheng M., Xie Z., Qu D., Li D., Du P., Jing X., Sun Z. (2013). On–off–on fluorescent carbon dot nanosensor for recognition of chromium (VI) and ascorbic acid based on the inner filter effect. ACS Appl. Mater. Interfaces.

[cit5] Wang D. F., Zhang G. L., Dai Z. Y., Zhou L. L., Bian P., Zheng K., Wu Z. Y., Cai D. Q. (2018). Sandwich-like Nanosystem for Simultaneous Removal of Cr(VI) and Cd(II) from Water and Soil. ACS Appl. Mater. Interfaces.

[cit6] Kailasa S. K., Koduru J. R., Desai M. L., Park T. J., Singhal R. K., Basu H. (2018). Recent progress on surface chemistry of plasmonic metal nanoparticles for colorimetric assay of drugs in pharmaceutical and biological samples. TrAC, Trends Anal. Chem..

[cit7] Costa M. (1997). Toxicity and carcinogenicity of Cr (VI) in animal models and humans. Crit. Rev. Toxicol..

[cit8] Gibb H. J., Lees P. S. J., Pinsky P. F., Rooney B. C. (2000). Lung cancer among workers in chromium chemical production. Am. J. Ind. Med..

[cit9] Ying Y. L., Liu Y., Wang X. Y., Mao Y. Y., Cao W., Hu P., Peng X. S. (2015). Two-Dimensional Titanium Carbide for Efficiently Reductive Removal of Highly Toxic Chromium(VI) from Water. ACS Appl. Mater. Interfaces.

[cit10] Li F. M., Liu J. M., Wang X. X., Lin L. P., Cai W. L., Lin X., Zeng Y. N., Li Z. M., Lin S. Q. (2011). Non-aggregation based label free colorimetric sensor for the detection of Cr (VI) based on selective etching of gold nanorods. Sens. Actuators, B.

[cit11] Derbyshire M., Lamberty A., Gardiner P. H. (1999). Optimization of the Simultaneous Determination of Cr(III) and Cr(VI) by Ion Chromatography with Chemiluminescence Detection. Anal. Chem..

[cit12] Zhang H. Y., Wang Y., Xiao S., Wang H., Wang J. H., Feng L. (2017). Rapid detection of Cr(VI) ions based on cobalt(II)-doped carbon dots. Biosens. Bioelectron..

[cit13] Zhong Y. L., Qiu X., Chen D. Y., Li N. J., Xu Q. F., Li H., He J. H., Lu J. M. (2016). Flexible Electrospun Carbon Nanofiber/Tin(IV) Sulfide Core/Sheath Membranes for Photocatalytically Treating Chromium(VI)-Containing Wastewater. ACS Appl. Mater. Interfaces.

[cit14] Zhang H.-Y., Wang Y., Xiao S., Wang H., Wang J.-H., Feng L. (2017). Rapid detection of Cr (VI) ions based on cobalt (II)-doped carbon dots. Biosens. Bioelectron..

[cit15] Zheng M., Xie Z., Qu D., Li D., Du P., Jing X., Sun Z. (2013). On-off-on fluorescent carbon dot nanosensor
for recognition of chromium (VI) and ascorbic acid based on the inner filter effect. ACS Appl. Mater. Interfaces.

[cit16] Toal S. J., Jones K. A., Magde D., Trogler W. C. (2005). Luminescent silole nanoparticles as chemoselective sensors for Cr(VI). J. Am. Chem. Soc..

[cit17] Yang Y., Wang G. Z., Deng Q., Ng D. H. L., Zhao H. J. (2014). Microwave-Assisted Fabrication of Nanoparticulate TiO_2_ Microspheres for Synergistic Photocatalytic Removal of Cr(VI) and Methyl Orange. ACS Appl. Mater. Interfaces.

[cit18] Phan L. M. T., Baek S. H., Nguyen T. P., Park K. Y., Ha S., Rafique R., Kailasa S. K., Park T. J. (2018). Synthesis of fluorescent silicon quantum dots for ultra-rapid and selective sensing of Cr(VI) ion and biomonitoring of cancer cells. Mater. Sci. Eng., C.

[cit19] Turyan I., Mandler D. (1997). Selective Determination of Cr(VI) by a Self-Assembled Monolayer-Based Electrode. Anal. Chem..

[cit20] Jin W., Yan K. (2015). Recent advances in electrochemical detection of toxic Cr(VI). RSC Adv..

[cit21] Tu J., Gan Y., Liang T., Wan H., Wang P. (2018). A miniaturized electrochemical system for high sensitive determination of chromium (VI) by screen-printed carbon electrode with gold nanoparticles modification. Sens. Actuators, B.

[cit22] Willets K. A., Van Duyne R. P. (2007). Localized surface plasmon resonance spectroscopy and sensing. Annu. Rev. Phys. Chem..

[cit23] Chen C. D., Cheng S. F., Chau L. K., Wang C. R. (2007). Sensing capability of the localized surface plasmon resonance of gold nanorods. Biosens. Bioelectron..

[cit24] Chen C. D., Cheng S. F., Chau L. K., Wang C. R. (2007). Sensing capability of the localized surface plasmon resonance of gold nanorods. Biosens. Bioelectron..

[cit25] Zhao J., Zhang X., Yonzon C. R., Haes A. J., Van Duyne R. P. (2006). Localized surface plasmon resonance biosensors. Nanomedicine.

[cit26] Homola J., Yee S. S., Gauglitz G. (1999). Surface plasmon resonance sensors. Sens. Actuators, B.

[cit27] Lou T., Chen L., Chen Z., Wang Y., Chen L., Li J. (2011). Colorimetric detection of trace copper ions based on catalytic leaching of silver-coated gold nanoparticles. ACS Appl. Mater. Interfaces.

[cit28] Barnes W. L., Dereux A., Ebbesen T. W. (2003). Surface plasmon subwavelength optics. Nature.

[cit29] Eustis S., El-Sayed M. A. (2006). Why gold nanoparticles are more precious than pretty gold: noble metal surface plasmon resonance and its enhancement of the radiative and nonradiative properties of nanocrystals of different shapes. Chem. Soc. Rev..

[cit30] Luther J. M., Jain P. K., Ewers T., Alivisatos A. P. (2011). Localized surface plasmon resonances arising from free carriers in doped quantum dots. Nat. Mater..

[cit31] Chen L., Li J., Chen L. (2014). Colorimetric detection of mercury species based on functionalized gold nanoparticles. ACS Appl. Mater. Interfaces.

[cit32] Chen L., Fu X., Lu W., Chen L. (2012). Highly sensitive and selective colorimetric sensing of Hg2+ based on the morphology transition of silver nanoprisms. ACS Appl. Mater. Interfaces.

[cit33] El-Sayed I. H., Huang X., El-Sayed M. A. (2005). Surface plasmon resonance scattering and absorption of anti-EGFR antibody conjugated gold nanoparticles in cancer diagnostics: applications
in oral cancer. Nano Lett..

[cit34] Hutter E., Fendler J. H. (2004). Exploitation of localized surface plasmon resonance. Adv. Mater..

[cit35] Masson J. F. (2017). Surface Plasmon Resonance Clinical Biosensors for Medical Diagnostics. ACS Sens..

[cit36] Schaadt D., Feng B., Yu E. (2005). Enhanced semiconductor optical absorption via surface plasmon excitation in metal nanoparticles. Appl. Phys. Lett..

[cit37] Haes A. J., Van Duyne R. P. (2002). A nanoscale optical biosensor: sensitivity and selectivity of an approach based on the localized surface plasmon resonance spectroscopy of triangular silver nanoparticles. J. Am. Chem. Soc..

[cit38] Saha K., Agasti S. S., Kim C., Li X., Rotello V. M. (2012). Gold nanoparticles in chemical and biological sensing. Chem. Rev..

[cit39] Li H., Chen D. X., Sun Y. L., Zheng Y. B., Tan L. L., Weiss P. S., Yang Y. W. (2013). Viologen-mediated assembly of and sensing with carboxylatopillar[5]arene-modified gold nanoparticles. J. Am. Chem. Soc..

[cit40] Lou T., Chen Z., Wang Y., Chen L. (2011). Blue-to-red colorimetric sensing strategy for Hg2+ and Ag+ via redox-regulated surface chemistry of gold nanoparticles. ACS Appl. Mater. Interfaces.

[cit41] Cao Y. C., Jin R., Mirkin C. A. (2002). Nanoparticles with Raman spectroscopic fingerprints for DNA and RNA detection. Science.

[cit42] Taton T. A., Mirkin C. A., Letsinger R. L. (2000). Scanometric DNA array detection with nanoparticle probes. Science.

[cit43] Yola M. L., Eren T., Atar N. (2015). A sensitive molecular imprinted electrochemical sensor based on gold nanoparticles decorated graphene oxide: Application to selective determination of tyrosine in milk. Sens. Actuators, B.

[cit44] Yu Y., Hong Y., Wang Y., Sun X., Liu B. (2017). Mecaptosuccinic acid modified gold nanoparticles as colorimetric sensor for fast detection and simultaneous identification of Cr3+. Sens. Actuators, B.

[cit45] Wang X., Wei Y., Wang S., Chen L. (2015). Red-to-blue colorimetric detection of chromium via Cr (III)-citrate chelating based on Tween 20-stabilized gold nanoparticles. Colloids Surf., A.

[cit46] Sugunan A., Thanachayanont C., Dutta J., Hilborn J. G. (2005). Heavy-metal ion sensors using chitosan-capped gold nanoparticles. Sci. Technol. Adv. Mater..

[cit47] Chen P., Yang F., Liao Q. X., Zhao Z. G., Zhang Y., Zhao P. P., Guo W. H., Bai R. X. (2017). Recycling and separation of rare earth resources lutetium from LYSO scraps using the diglycol amic acid functional XAD-type resin. Waste Manage..

[cit48] Bai R. X., Yang F., Zhang Y., Zhao Z. G., Liao Q. X., Chen P., Zhao P. P., Guo W. H., Cai C. Q. (2018). Preparation of elastic diglycolamic-acid modified chitosan sponges and their application to recycling of rare-earth from waste phosphor powder. Carbohydr. Polym..

[cit49] Bai R. X., Zhang Y., Zhao Z. G., Liao Q. X., Chen P., Zhao P. P., Guo W. H., Yang F., Li L. C. (2018). Rapid and highly selective removal of lead in simulated wastewater of rare-earth industry using diglycolamic-acid functionalized magnetic chitosan adsorbents. J. Ind. Eng. Chem..

[cit50] Yang F., Baba Y., Kubota F., Kamiya N., Goto M. (2012). Extraction and Separation of Rare Earth Metal Ions with DODGAA in Ionic liquids. Solvent Extr. Res. Dev., Jpn..

[cit51] Baba Y., Fukami A., Kubota F., Kamiya N., Goto M. (2014). Selective extraction of scandium from yttrium and lanthanides with amic acid-type extractant containing alkylamide and glycine moieties. RSC Adv..

[cit52] Schroeder D. C., Lee G. F. (1975). Potential Transformations of Chromium in Natural-Waters. Water, Air, Soil Pollut..

[cit53] Cranston R., Murray J. (1978). The determination of chromium species in natural waters. Anal. Chim. Acta.

[cit54] Kumar P. S., Kirthika K., Kumar K. S. (2008). Removal of hexavalent chromium ions from aqueous solutions by an anion-exchange resin. Adsorpt. Sci. Technol..

[cit55] Korak J. A., Huggins R., Arias-Paic M. (2017). Regeneration of pilot-scale ion exchange columns for hexavalent chromium removal. Water Res..

[cit56] Qdais H. A., Moussa H. (2004). Removal of heavy metals from wastewater by membrane processes: a comparative study. Desalination.

[cit57] Kozlowski C. A., Walkowiak W. (2002). Removal of chromium(VI) from aqueous solutions by polymer inclusion membranes. Water Res..

[cit58] Burgess J. E., Strong P. J. (2008). Treatment Methods for Wine-Related and Distillery Wastewaters: A Review AU. Biorem. J..

[cit59] Andreottola G., Foladori P., Ziglio G. (2009). Biological treatment of winery wastewater: an overview. Water Sci. Technol..

[cit60] Dubrawski K. L., Du C., Mohseni M. (2014). General Potential-Current Model and Validation for Electrocoagulation. Electrochim. Acta.

[cit61] Meas Y., Ramirez J. A., Villalon M. A., Chapman T. W. (2010). Industrial wastewaters treated by electrocoagulation. Electrochim. Acta.

[cit62] Barrera-Díaz C. E., Lugo-Lugo V., Bilyeu B. (2012). A review of chemical, electrochemical and biological methods for aqueous Cr(VI) reduction. J. Hazard. Mater..

[cit63] Zhong D., Zhang Y., Wang L., Chen J., Jiang Y., Tsang D. C., Zhao Z., Ren S., Liu Z., Crittenden J. C. (2018). Mechanistic insights into adsorption and reduction of hexavalent chromium from water using magnetic biochar composite: Key roles of Fe3O4 and persistent free radicals. Environ. Pollut..

