# Supplementary material for: Highly selective and sensitive probes for the detection of Cr(vi) in aqueous solutions using diglycolic acid-functionalized Au nanoparticles
Source: RSC Adv. 2019 Apr 8;9(19):10958–65. doi: 10.1039/c9ra00010k (PMC9062733; doi:10.1039/c9ra00010k)
Supplement: RA-009-C9RA00010K-s001 [file RA-009-C9RA00010K-s001.pdf]

Electronic Supplementary Material (ESI) for RSC Advances.
This journal is © The Royal Society of Chemistry 2019

Supplementary information of

Highly selective and sensitive probes for the detection of Cr(VI) in aqueous solutions using diglycolamic acid functionalized Au nanoparticles

Authors: Yang Zhang[1,2,*], Ruixi Bai[1,2], Zhigang Zhao[1,2], Qiuxia Liao[1,2], Peng Chen[1,2], Wanghuan Guo[1,2], Chunqing Cai[1,2], and Fan Yang[1,2,*]

[1]CAS Key Laboratory of Design and Assembly of Functional Nanostructures and Fujian Provincial Key Laboratory of Nanomaterials, Fujian Institute of Research on the Structure of Matter, Chinese Academy of Sciences

[2]Xiamen Institute of Rare Earth Materials, Haixi Institute, Chinese Academy of Sciences, Xiamen 361021

* Corresponding author

Dr. Yang Zhang

E-mail: yzhang@fjirsm.ac.cn;

Prof. Fan Yang

E-mail: fanyang2013@fjirsm.ac.cn;

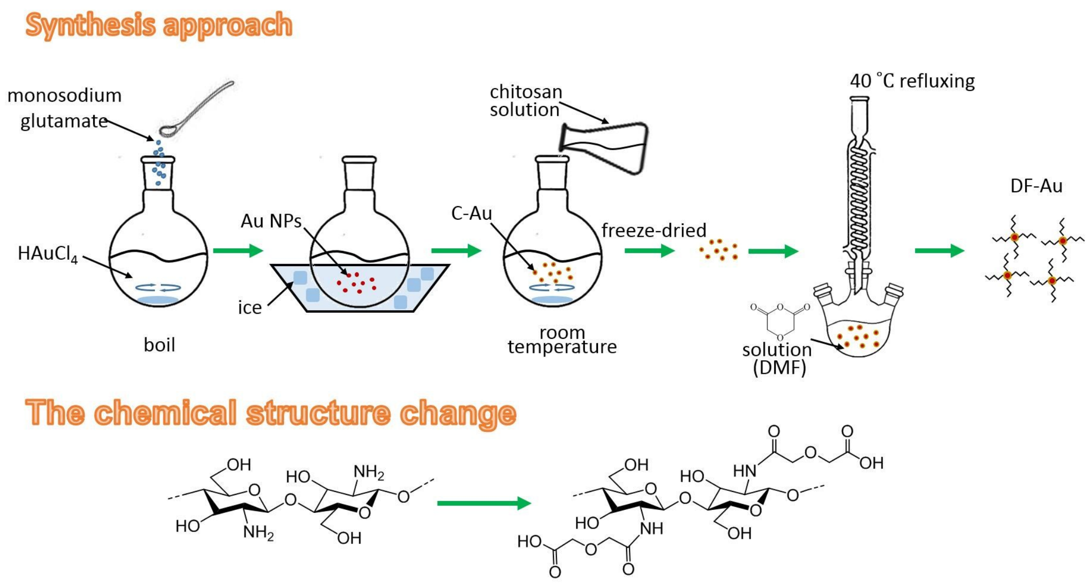

Scheme S1. The flow chart of the synthesis process of the DF-Au sensors.
